# Identifying the Driving Factors of Water Quality in a Sub-Watershed of the Republican River Basin, Kansas USA

**DOI:** 10.3390/ijerph15051041

**Published:** 2018-05-22

**Authors:** Morgen W. V. Burke, Mojtaba Shahabi, Yeqian Xu, Haochi Zheng, Xiaodong Zhang, Jeffrey VanLooy

**Affiliations:** Department of Earth System Science and Policy, University of North Dakota, Grand Forks, ND 58202, USA; morgen.burke@und.edu (M.W.V.B.); seyedmojtaba.shahabi@und.edu (M.S.); yeqian.xu@und.edu (Y.X.); zhang@aero.und.edu (X.Z.); jvanlooy@aero.und.edu (J.V.)

**Keywords:** nonpoint source pollution, regression, land use change

## Abstract

Studies have shown that the agricultural expansion and land use changes in the Midwest of the U.S. are major drivers for increased nonpoint source pollution throughout the regional river systems. In this study, we empirically examined the relationship of planted area and production of three dominant crops with nitrate flux in the Republican River, Kansas, a sub-watershed of Mississippi River Basin. Our results show that land use in the region could not explain the observed changes in nitrate flux in the river. Instead, after including explanatory variables such as precipitation, growing degree days, and well water irrigation in the regression model we found that irrigation and spring precipitation could explain >85% of the variability in nitrate flux from 2000 to 2014. This suggests that changes in crop acreage and production alone cannot explain variability in nitrate flux. Future agricultural policy for the region should focus on controlling both the timing and amount of fertilizer applied to the field to reduce the potential leaching of excess fertilizer through spring time runoff and/or over-irrigation into nearby river systems.

## 1. Introduction

Nonpoint source (NPS) pollution continues to be a major source of water quality impairment in the U.S. In 2017, the U.S. Environmental Protection Agency (EPA) reported that NPS from agriculture was one of the most common sources of pollutants for streams and lakes [1]. Agricultural production practices often result in over-application of fertilizers, which can cause leaching of excess nitrogen and phosphorous into nearby aquatic ecosystems through runoff [2]. Carpenter et al. [3] estimated an average of 44 kg/ha yr of phosphorous is applied for agricultural production in the U.S. but only 18 kg/ha yr is harvested, resulting in an estimated 2% to 30% of phosphorous applied to cropland potentially leaching into nearby surface water. In freshwater and marine environments, excessive nutrients can increase the level of primary productivity and cause eutrophication [2]. Within the Mississippi River Basin, many of these pollutants make their way to the coast and end up in the Gulf of Mexico. Nitrogen concentrations in the Gulf of Mexico have more than tripled since 1970 [4], and the hypoxic conditions caused by the increased nitrogen loads continue to impair the gulf’s ecosystems [5,6].

Changes in agricultural policies and practices, and the types of crops grown within a watershed have been found to influence the nitrogen inputs to the environment [7]. Land use change in the Mississippi River Basin has been identified as a main driver causing increased nitrogen in streams and rivers [8,9,10]. Broussard and Turner [10] examined changes in nitrogen concentration across various watersheds within the Mississippi River Basin, and found that between the two study periods, 1906–1912 and 1993–1997, mean river nitrate concentrations had tripled, going from 0.60 mg/L to 1.79 mg/L. 

The Republican River Basin (RRB) is within a region of the U.S. that has seen a decrease in crop diversity as corn, soybeans and wheat went from making up 46% of cropland in the 1950s to 61% by 1992 [11]. It has been found that changes in land use practices can lead to increased river discharge [12]. In addition, the expansion of corn and soybeans has been related to greater levels of nitrates leaching into nearby aquatic systems [9,13]. While several papers have investigated agricultural watersheds that have seen a significant increase in NPS pollution [7,8,9,10,11,12], investigating a watershed that has not changed offers a new perspective. Understanding why the Republican River has not experienced similar increases in nutrient concentration can provide insights for land management, which may be applicable for controlling NPS pollution within agricultural regions.

While physically-based hydrologic models can simulate hydrological processes in a watershed, they often require a large amount of data that may not be available and make various assumptions that are difficult to validate for the study area [14]. Statistical analysis, on the other hand, can provide a simple yet effective examination based on observed information. The objective of this study was to empirically investigate the relationship between agricultural land use change and water quality in the RRB from 2000 to 2014. To remove potential error caused by changes in river base flow, we used nitrate flux instead of concentration to measure water quality and examined land use by focusing on the major crops grown in the region [13]. We hypothesized that cropland and crop production are statistically correlated with nitrate flux in the river, particularly for corn, winter wheat, and soybeans.

## 2. Materials and Methods

### 2.1. Study Area Selection

**Figure 1:**
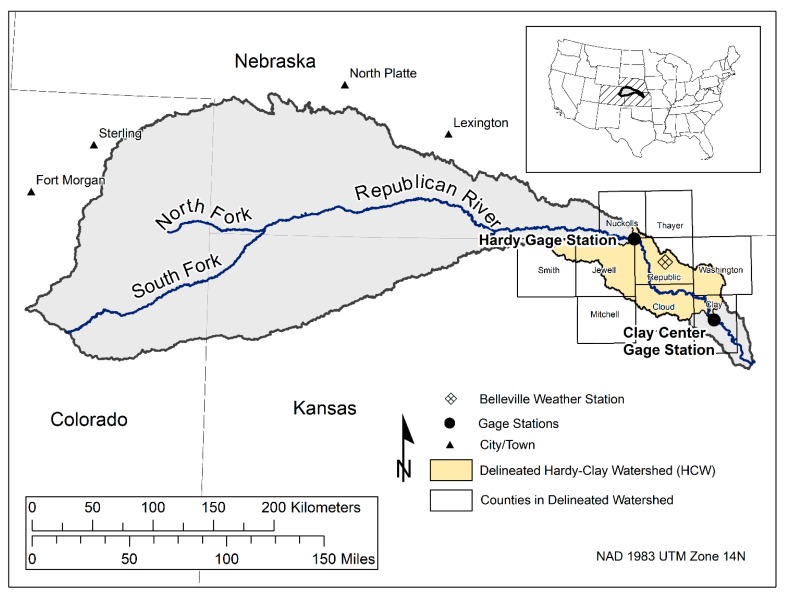
The Republican River Basin with the delineated watershed between the Hardy and Clay Center gage stations, the Hardy-Clay Watershed (HCW). Also shown is the location of the Belleville weather station, and the boundary of the counties that contain part of the HCW.

The RRB (Figure 1) is centrally located within the Contiguous U.S., and covers part of the three states: Nebraska, Colorado, and Kansas. Within the river basin, defined by the U.S. Geological Survey (USGS) Hydrological Unit Code (HUC) 102500, there are approximately 6.45 million hectares of land (15.95 million acres), among which approximately 3.1 million hectares (7.7 million acres) were crop land based on the 2012 estimates. In 2012, the RRB had approximately 11,649 farms, with an average farm size of 504 hectares (1246 acres), which is 329 hectares (813 acres) larger than the national average [15]. The region is known for its extremely complex water management system and decreased water supply resulting in water-use disputes among the three agricultural states [16].

To examine how land use varies across the Republican River, we used ArcGIS Desktop 10.3.1 (Environmental Systems Research Institute, Redlands, CA, USA) to clip the U.S. Department of Agriculture (USDA) Cropland Data Layer (CDL) [17] to the boundary of the RRB. The land cover information of the three RRB states is available annually starting in 2008 and contains 66 different land cover categories in the CDL. Among these 66 categories, six make up more than 90% of the land cover classes: corn, soybeans, winter wheat, grassland/pasture, fallow/idle cropland and sorghum.

The major agricultural crops in the RRB are corn, soybeans, and winter wheat, making up approximately 70% of all cropland. Corn is planted between early April and late May, while soybeans are planted between early May and late June. Both are harvested between early September and late November. Winter wheat is planted from early September to late October and harvested between the middle of June to late July of the following year. All three crops are planted and harvested during similar periods within the states surrounding the RRB [18]. In Kansas 70.4% of corn acres receive nitrogen fertilizer in the spring time before planting, and 34.5% receive nitrogen during planting; 39.4% of soybean acres receive nitrogen in the spring time before planting, and 64.3% of acres receive nitrogen during planting; 59.3% of acres of winter wheat receive nitrogen fertilizer in the fall before planting, and 29.9% receive nitrogen during planting [19].

For our study, we selected the area between the Hardy gage (USGS 06853500) and Clay Center gage (USGS 06856600), referred to hereafter as the Hardy-Clay Watershed (HCW) (Figure 1). This site was chosen as it does not contain any man-made water control structures, such as reservoirs which can be found at several other locations upstream and downstream of the Republic River. We delineated this portion of the watershed based on a USGS digital elevation model (DEM) using the Spatial Analyst Hydrology toolset (Environmental Systems Research Institute, Redlands, CA, USA) within ArcGIS Desktop 10.3.1 to find the catchment area between the two gage stations. The DEM has a spatial resolution of 1/9 arc second, and was collected in January 2013. The delineated area covers 513,162 hectares of land (1.27 million acres) making up approximately 8% of the RRB.

### 2.2. Land Cover Area, Cropland Area and Production Calculations

**Figure 2:**
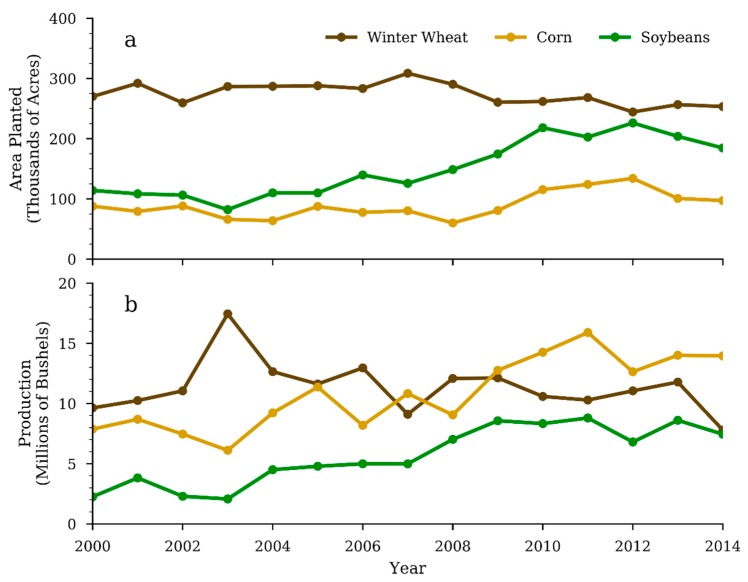
The annual planted acreage (**a**) and production in bushels (**b**) for corn, winter wheat, and soybeans in the HCW.

Using the CDL from 2006 to 2014, we identified 22 land cover classes within the HCW and estimated the total area for each of them. Among those identified land classes, which include various crops, developed land, wetlands, forests, and grassland, corn, winter wheat and soybeans are of particular interest because they make up approximately 80% of cropland in the HCW. To obtain the spatial information of the cropland from 2000 to 2006 that is not available from the CDL we adopted the county level annual crop planted area and total production data from the USDA National Agricultural Statistic Service (NASS) for the three major crops. We further adjusted these numbers for the study region based on the percentage of HCW area contained within the county by assuming that crops are evenly distributed within each county. This process extended both crop production information for these three crops and their acreage information from 9 (2006–2014) to 15 (2000–2014) years (Figure 2). 

Examining Figure 2, we can identify the changes in the three major crops over the study period. In 2000, winter wheat was the main crop for the study area with 270,083 acres planted and 9.6 million bushels produced. Corn and soybean had 87,802 and 113,967 acres planted, and 7.9 and 3.8 million bushels produced, respectively. However, by 2014 corn became the main crop in the region in terms of production, increasing nearly 80% and reaching 14.0 million bushels, even though the planted acres increased by only 10%. Over the same time, the planted acres of soybeans had doubled and the total production had more than tripled, while winter wheat had seen a decrease with 253,286 acres planted and 7.8 million bushels produced. 

**Figure 3:**
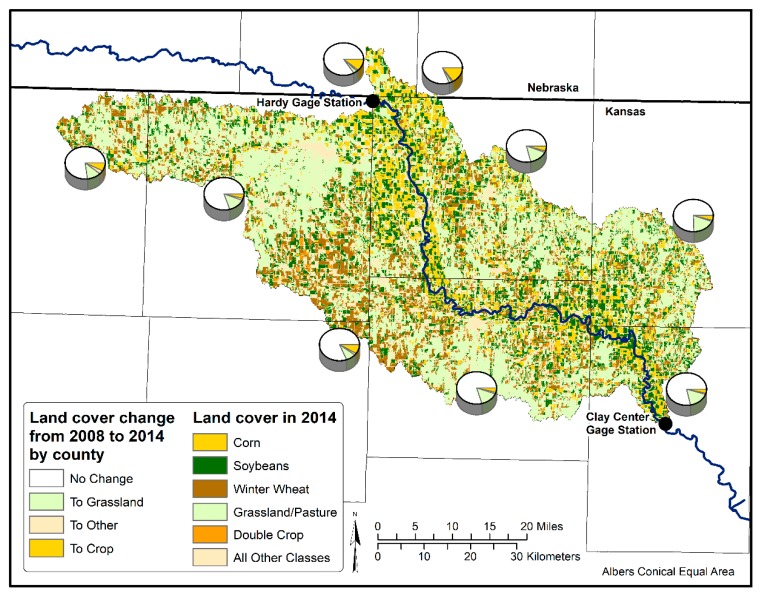
The 2014 Cropland Data Layer (CDL), highlighting the three main crops of corn, soybeans, and winter wheat, as well as grassland/pasture and double cropped fields in the HCW. The pie charts show the county-level average change among three major land classes between year 2008 and year 2014. The three major land classes are: the Grassland class representing the grassland/pasture land covers, the Crop class representing all agricultural crops, and the other class representing all remaining land covers, for example, urban areas, roads, forests, wetlands and open water.

Grouping CDL land covers in the HCW into three classes: the Grassland class represents the Grassland/Pasture class; the Crop class represents all agricultural crops; the Other class includes all remaining classes, such as urban areas, roads, forests, wetlands and open water, we estimated the changes in these three major land covers between year 2008 and year 2014 (Figure 3). Change to cropland mainly occurred in the north and west portions of the watershed, and only sporadic land use change to cropland was detected in the rest of the study area. The increases of corn and soybeans seen in Figure 2 are likely due to conversions of small crops, which we found had decreased by 40.8% during the study period, rather than expansion into the grassland/pasture and other land use classes. Overall, between 2008 and 2014, grassland/pasture increased area by 11.7%, while the cropland and the other land class categories decreased by 6.4% and 5.3% respectively.

### 2.3. Weather Data—Precipitation and Growing Degree Days

Weather data recorded at station GHCND: USC00140682 located near Belleville, KS (Figure 1) was acquired from the National Oceanic and Atmospheric Administration’s (NOAA) National Center for Environmental Information (NCEI). We downloaded daily data of maximum and minimum temperature (°C), precipitation (mm) and snowfall depth (mm) from 1 November 1999 to 31 December 2014.

**Figure 4:**
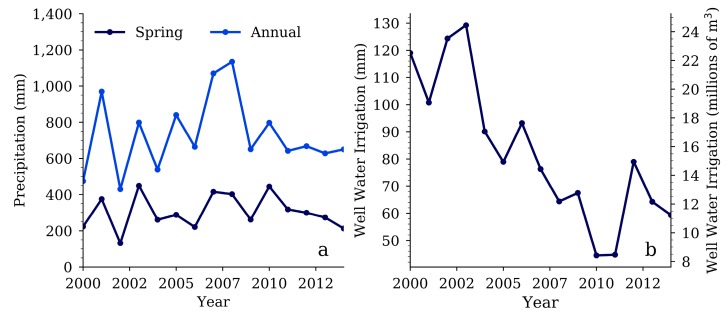
(**a**) Spring and annual precipitation data, and (**b**) annual total well water irrigation.

The daily weather data was further broken up into three seasons: Fall/Winter from November to February, Spring from March to June, and Summer from July to October. This regrouping can better represent different weather patterns over the year and align with the different growing seasons of the crops. Corn and soybeans are planted during the spring, and harvested in the late summer to early fall, while winter wheat is planted in the fall/winter and harvested in the spring. We calculated accumulated precipitation (Figure 4a) and snowfall for each of the seasons.

From 2000 to 2014, the spring and summer seasons typically have the most precipitation with an average of 305 mm and 332 mm respectively, while the fall/winter season always has the least with an average of 94 mm. Snowfall depth is always greatest during the fall/winter season with an average of 393 mm compared to the average spring snowfall of 41 mm. In 2001 and 2004, the accumulated snowfall for the fall/winter season was 1012 mm and 820 mm, respectively, more than double the average.

To estimate the accumulated heat for crop development, growing degree days (GDD) were calculated for each of the three seasons and the base level temperature (*Tbase*) was set at (0, 10) °C (Equation (1)). Typical *Tbase* values are 0 °C for soybeans and winter wheat and 10 °C for corn in the Great Plains region [20].

(1) $$\mathrm{GDD}\;=\frac{Tmax\;+\;Tmin}{2}-Tbase,\;\;\;\;\;if\;\frac{Tmax\;+\;Tmin}{2}>Tbase\;\mathrm{GDD}=0,\;\;\;otherwise$$

Given the definition of GDD, the base level of 0 °C always had the greatest number of accumulated GDD. Among the seasons, summer typically had the most GDD, with spring in the middle, and fall/winter having the least. This was expected for the study area. The GDDs estimated with two base levels were all highly correlated with each other for any given season.

### 2.4. Well Water Irrigation

We acquired well water irrigation usage information from the Water Information Management and Analysis System jointly operated by the Kansas Department of Agriculture’s Division of Water Resources and the Kansas Geological Survey. Within the HCW we found 380 wells that have reported annual water usage for irrigation. The annual well water usage data for each well was summed together to provide total annual irrigation (AF). To make these annual volumetric values comparable with the precipitation data, we divided them by the annual total irrigated acres of corn, soybeans, and winter wheat reported by the NASS. From 2000 to 2014, well water usage follows a decreasing trend, with the first 4 years reporting usage above 100 mm, and the last four years reporting usage below 80 mm (Figure 4b). On average 82.3 mm of well water was used for irrigation annually.

### 2.5. Estimating Nitrate Flux

To remove potential error caused by changes in river base flow, we used nitrate flux (kg/year) instead of concentration (mg/L) as a measure of water quality [8,10]. We retrieved the data on nitrate concentration (mg/L) and river discharge (L/s) recorded for the Hardy (USGS 06853500) and Clay Center (USGS 06856600) gage stations from 1999 to 2014 through the USGS National Water Information System web interface. In total, only 150 nitrate concentration measurements were available for the two gage stations over the 15 year period; as a common approach, gaps in the available data were interpolated [13]. We used the Load Estimator model (LOADEST) [21] implemented by the USGS LoadRunner software [22] to estimate the average daily nitrate flux (kg/day) for each month using the measured data. The USGS Load Runner software has 9 possible variations for the LOADEST model, and the best variation is selected based on the Schwarz Posterior Probability Criteria (SPPC). For the Hardy gage station, we selected model 5 with an SPPC of −93.437. For the Clay Center gage station, we selected model 4 with an SPPC of −79.974 [22]. In calculating the nitrate flux at each of the two gage stations, all nitrate concentration values outside of 3 standard deviations for the gage station dataset were excluded from the model and gaps of up to 7 days were allowed in the data before a day would not be calculated. Using the daily averaged nitrate flux (kg/day) for each month, we calculated nitrate flux for monthly (kg/month) and annual (kg/year) totals from January 1999 to December 2014 [22]. We further subtracted the annual upstream Hardy gage station nitrate flux from the downstream Clay Center gage station nitrate flux to remove the base flow and obtain the nitrate flux data that captures the runoff only occurring within the HCW. We also used measured nitrate flux data to verify the accuracy of the interpolated values from the LOADEST model.

**Figure 5:**
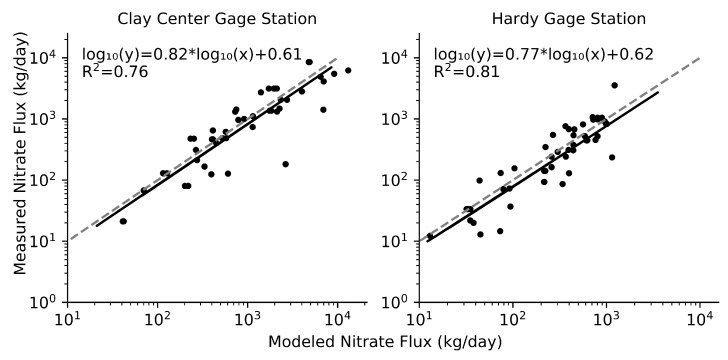
LoadRunner model evaluation using a log_10_ transformation of both measured and modeled nitrate flux values. For each gage station 50 measured data points are compared against the values predicted by the LOADEST Model (black line), the grey-dashed line represents a perfect 1:1 fit.

Figure 5 compares nitrate flux values predicted by the LOADEST model against the measurements at the two gage stations. To test the accuracy of the LOADEST model in predicting nitrate flux 10 random samples were set aside for each gage station before processing the model with the remaining water quality data. This was repeated with 5 permutations to collect a total of 50 sample points for each gage station for model validation. The comparison shown in Figure 5 indicates that the LOADEST model performed well, with an R^2^ of 0.76 and 0.81 for Clay Center and Hardy gage stations, respectively.

**Figure 6:**
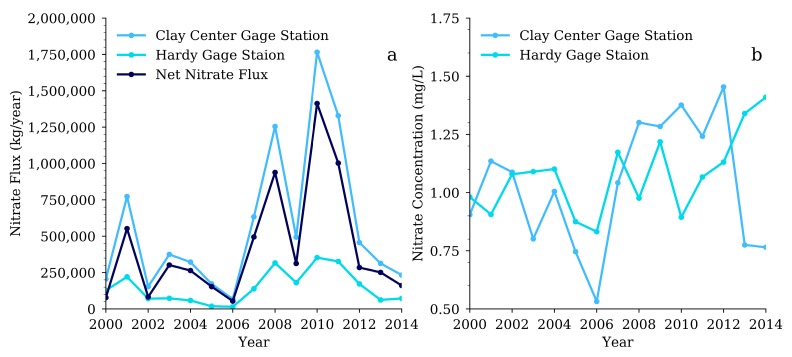
(**a**) Nitrate flux estimated for the two USGS gage stations, Hardy and Clay Center and the net nitrate flux for the HCW, which was calculated as the difference between Hardy and Clay Center gage stations; (**b**) The annual average nitrate concentrations (mg/L) estimated by the LOADEST model for the two USGS gage stations. USGS: U.S. Geological Survey

Using the LOADEST model tested in Figure 5, but now including all water quality measurements, the annual nitrate fluxes for the two gage stations were calculated from 2000 to 2014, and the difference between the two stations represents the net nitrate flux for the HCW (Figure 6a). During the study period, the upstream Hardy gage station had a consistently lower nitrate flux than the downstream Clay Center gage station. Large increases in nitrate flux are seen in 2001, 2008, 2010, and 2011 at the Clay Center gage station that did not appear at the upstream Hardy gage station.

### 2.6. Statistical Model

We conducted linear regression analyses using the software R 3.3.3 (R Foundation for Statistical Computing, Vienna, Austria) to examine the relationship of land uses and weather, against nitrate flux. We tested each of the independent variables for correlation against the annual nitrate flux, and then we brought variables identified as significant (*p* < 0.10) into the multilinear regression model. We further tested for multicollinearity among all explanatory variables used in the regression analysis [23]. If we found any two variables to be closely correlated (*p* < 0.05), we then removed the less significant one from the model.

While the cropland and irrigation data used in the annual regression model only consisted of a single data point per year, the weather data is available as a daily record and can be aggregated to produce monthly and seasonal values. Thus, additional analyses were done with monthly and seasonally accumulated precipitation, snowfall depth and GDD to examine their relationships with corresponding monthly and seasonal nitrate flux.

## 3. Results

To test the original hypothesis, we assessed the relationship between the 22 CDL land cover classes and nitrate flux. Unexpectedly, neither the acreage nor the production of the three major crops had any significant relationship with the annual nitrate flux. The only CDL land cover class that showed significance was open water. We then conducted regression analysis with the precipitation, temperature and irrigation datasets. Table A1 reports the descriptive statistics and the regression results of all independent variables assessed in the linear regressions against the annual nitrate flux data. From the linear regression analyses, we identified four significant variables (*p* < 0.10), the GDD for the fall/winter season with base level 0 °C, spring precipitation, well water irrigation, and open water (Table 1).

**Figure 7:**
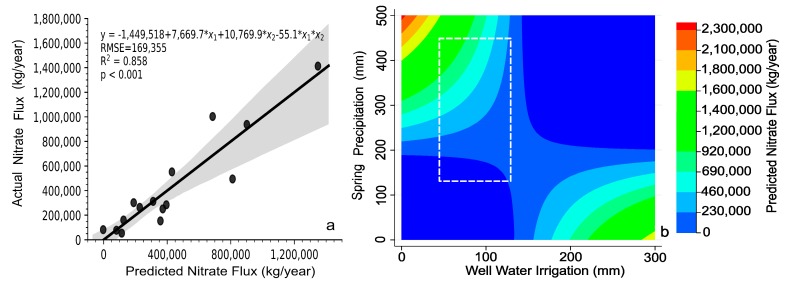
(**a**) Comparison of annual nitrate flux predicted by the multilinear regression model (spring precipitation, well water irrigation and their interaction) and measured; (**b**) Contour plot of the model as functions of spring precipitation and annual well irrigation. The dashed white box denotes the ranges of values of the two variables used in regression.

These four significant variables were then included as the explanatory variables in a multiple-variable linear regression analysis. However, the results indicated that the coefficient estimates of GDD and open water were no longer significant and hence were removed from the model. This left spring precipitation and well water irrigation as the only remaining explanatory variables. Although the two were not statistically correlated in our data (*p* = 0.40), the decision to use irrigation is often based on the amount of precipitation that falls on the fields. Consequently, the marginal effect of irrigation on nitrate flux is also dependent on precipitation. To capture this indirect impact of an agricultural producer’s decision-making, we added in the model an interaction term between spring precipitation and irrigation, both being main water supply sources. The final model is shown in Table 2 and its performance is examined in Figure 7. Both spring precipitation and well water irrigation show a positive impact on annual nitrate flux. Among the three explanatory variables, spring precipitation had the greatest influence on model performance (*p* < 0.001), though the interaction term was also highly significant (*p* = 0.004). Nearly 86% of variability in the annual nitrate flux can be explained by the model (Figure 7a), with spring precipitation and well water irrigation each explaining 47% and 38% of the variability, respectively (Table 1).

## 4. Discussion

This study originally aimed to examine land use change, measured as the expansion of agricultural crops in the HCW, and its role in affecting water quality. However, our analysis showed that except for the indirect impacts through irrigation practice, the agricultural expansion itself in the HCW did not have a significant relationship with nitrate flux in the river. Broussard and Turner [10] found a direct relationship between the nitrate concentration and both the percentage of corn cropland area and the corn production. However, their study included multiple watersheds across parts of the Midwestern U.S. that contain heavy agricultural expansion. In contrast to the substantial increase in planted corn in areas such as Iowa and Illinois, the HCW has seen only 10% increases in corn acreage (Figure 2). Our analysis showed that the land cover data had little explanatory power in the nitrate flux, to which no statistically significant relationship exists for either the area of crops planted, or the amount of crop production taking place, or the area of wetlands, grasslands/pasture, forest or developed areas (Table A1). Therefore, we had to reject the initial hypothesis. 

Broussard and Turner [10] found in their study of the Mississippi Watershed that the RRB had a nitrate concentration of roughly 1 mg/L from 1906 to 1912 and a similar concentration again from 1993 to 1997. This is consistent with what we found in the two USGS gage station data with nitrate concentrations ranging from less than 0.5 mg/L to almost 3 mg/L during our study period (Figure 6b). It is surprising to see such low levels of nitrates in the RRB when areas with similar agricultural practices in Iowa have watersheds with nitrate concentrations ranging from 3.0 to 10.5 mg/L [8]. The HCW is right on the edge of the region that had experienced large increases in production of corn and soybeans between the 1960s and the late 1990s, and may not have experienced as much growth and intensification as parts of Iowa [9]. In 2012, Kansas had about 15.5 million more acres of farmland than Iowa, and planted 9.0 million more acres of winter wheat, but Iowa had 9.7 million more acres of corn and 5.7 million more acres of soybeans than Kansas [15]. Not only does Iowa have more corn and soybeans than Kansas, it has less available farmland, meaning the corn and soybeans are planted at greater densities within the region. The lower density of corn and soybean crops seen in the HCW, often separated by grassland/pasture (Figure 3) might explain, at least partly, why the nitrate concentrations in the Republic River are lower than expected.

**Figure 8:**
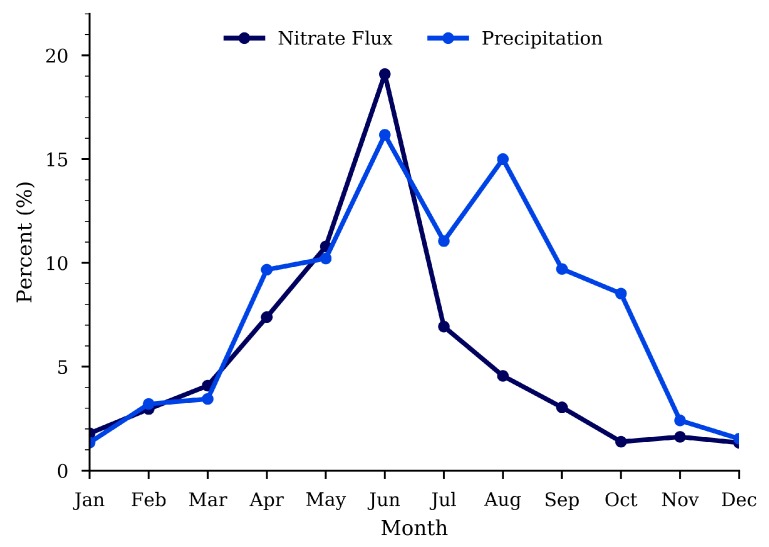
Median percentage contributions from 2000 to 2014 of each month to the annual value for nitrate flux (dark blue) and precipitation (light blue).

In the absence of a significant effect due to land use change, the multilinear regression analysis detected spring precipitation and well water irrigation as the two driving factors affecting the amount of nitrates ending up in the river. The final model (Figure 7) showed that spring precipitation and well water irrigation can explain 86% of variability in annual nitrate flux in the study area, indicating a strong relationship between runoff from spring precipitation and irrigation, and the amount of nitrates ending up in the river channel on an annual basis. This is not surprising given the farming practices in the study area. Nitrogen application for corn primarily takes place in the spring time, when nitrogen is more accessible to winter snowmelt and spring precipitation [9]. For winter wheat, nitrogen is typically applied late in the fall during a time of lower precipitation (Figure 8) and could become accessible to runoff in the early spring [19]. Once crops have matured, nitrogen is bound by the crops, and less available to become dissolved in runoff [24].

The LOADEST model used to predict nitrate flux for our study area depends heavily on information about the water quantity. Because of this, the rate of flow, made up of both base flow and surface inputs, has a great influence on the total amount of nitrates in the water and on the amount of nitrate flux at any given time. For example, a decrease in precipitation lowers the input of water to the Republican River, consequently lowering the rivers rate of flow and resulting in a lower amount of nitrate flux. The Republican River within the HCW has a history of having a low water quantity after much of the water is removed for irrigation of agricultural crops upstream. Removing large volumes of the base flow from the river every year for irrigation would reduce the total amount of nitrate flux during periods of high irrigation. Kansas filled a lawsuit against Nebraska for overuse of the Republican River water supply in 2006 [25], and this year was found to have relatively low nitrate flux levels (Figure 6). This is likely not a coincidence, but instead evidence showing the relationship between quantity of water and nitrate flux [9].

**Figure A1:**
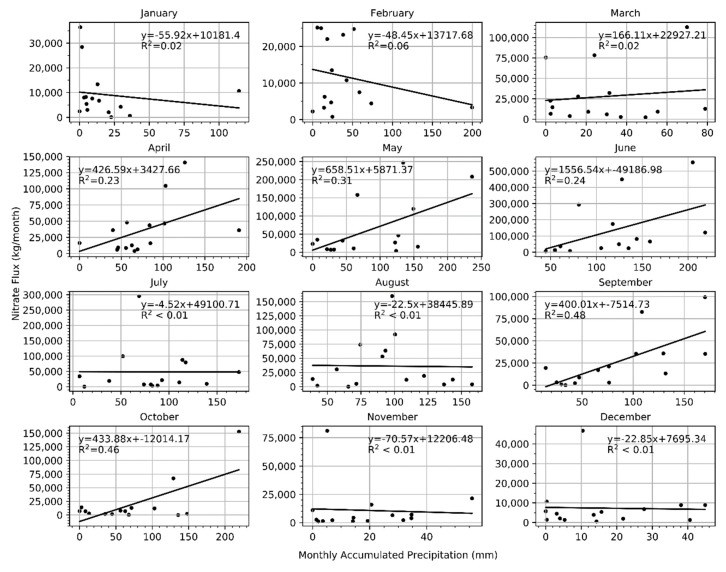
The relationship between the mean monthly nitrate flux for a given month and the total precipitation for that month.

We further examined the correlation between monthly nitrate flux (kg/month) in the HCW and monthly precipitation. The monthly precipitation in April, May, and June yield an R^2^ of 0.23, 0.31, and 0.24 respectively and in September and October yield an R^2^ of 0.48 and 0.46 respectively (Figure A1). Donner et al. [9] examined the U.S. Midwest and also found a strong relationship between nitrate leaching and precipitation in March, April, and May. However, they did not find a strong relationship in the fall months. This monthly relationship that we found in September and October may be related to the fact that approximately 60% of winter wheat acres are fertilized during these two months [19]. However, the estimated impact of Fall/Winter precipitation on annual nitrate flux was not statistically significant with *p* = 0.258 and R^2^ = 0.097. This is because that while precipitation and nitrate flux in the fall may follow a similar pattern, their fractional contributions are negligible when compared with the spring time nitrate flux. On average from 2000 to 2014, September and October make up only 3.0% and 1.4%, while March, April, May, and June make up 4.1%, 7.3%, 10.8% and 19.0% of the annual nitrate flux (Figure 8).

As a supplementary water supply source, well water irrigation positively correlated with annual nitrate flux (*p* = 0.07; Table 2), suggesting that an increase in irrigation increases nitrate leaching as well. Our model also includes an interaction term between precipitation and irrigation (*p* = 0.004) with a negative coefficient estimated. This reflects the fact that agricultural producers tend to use more well water to meet irrigation needs during times of lower precipitation. The model was fitted using the data whose values fall within the bounds defined by a dashed white box in Figure 7b. Extrapolating the model into both lower and higher values reveals some interesting points. First, the impact on nitrate flux becomes important when spring precipitation is >200 mm, below which the impact remains low. Second, at the present level of irrigation, for which the maximum amount of water withdraw is <130 mm (or equivalent of 2.46 × 10^7^ m^3^), nitrate leaching due to irrigation alone is minimal. However, if annual irrigation exceeds the threshold of 150 mm (~2.84 × 10^7^ m^3^), nitrate flux will start to increase with further irrigation water usage. Third, the model also predicts a decrease of nitrate flux when both precipitation and irrigation increase, which does not make physical sense. While mathematically possible, it is highly unlikely and uneconomical for farmers to choose irrigation when there is an excess of precipitation. Therefore, the high-precipitation and high-irrigation domain under which the model would fail is very unlikely to occur in practice.

Focused recommendations based on our results can be made for parts of Kansas including the HCW and other agricultural areas in the Midwestern U.S. that have highly variable precipitation. In these areas, one strategy for reducing nitrate leaching is to adjust the timing of fertilization for agriculture so that the soil and fertilizer is not as easily accessible to spring time precipitation. This could mean applying smaller amounts of fertilizer throughout different parts of the season when the plants need it, and not all at once when crops are planted in the spring. It could also mean using precision agriculture to only apply the amount of fertilizer needed in specific parts of the field to reduce the chance of over fertilization [24,26,27]. In addition, using irrigation only when needed, and not irrigating beyond the threshold value (which is ~150 mm in the HCW but likely varies in other watersheds), should prevent nitrate leaching through irrigation runoff. Strategic placing of crops in the watershed based on each crop’s nutrient uptake efficiency could also act as a nutrient removal strategy. In the RRB the grassland/pasture land cover may help decrease the connectivity between agricultural crops and nearby river channels. A similar management practice can be done with the placement of riparian buffers to filter nutrients from runoff before they enter a stream or river [28].

## 5. Conclusions

Our study found a strong relationship between springtime precipitation and well water irrigation, and nitrate flux for the HCW. Based on multilinear regression analysis, we did not found that change in land use has a significant effect on nitrate flux in the Republican River within the HCW. The examination of a watershed that has historically low concentrations of nitrates, allowed us to gain new insights into the relationships between water quality and the quantity of surface water inputs within an agricultural watershed. Future work for this study can include testing the relationships in other parts of the RRB. We chose the study area based on available data and a lack of reservoirs and other water control structures between the gage stations. Other areas of the RRB may fall under these restrictions. Alternatively, the same study area can be reexamined later when future gage station information is available. Our results suggest that other drivers of NPS pollution need to be considered for policy implementation. Better management practice can be made to reduce nitrate loading in the watershed by reducing agricultural fertilization during spring time with high precipitation, avoiding over application of fertilizer beyond the needs of the crops, and avoiding excessive amounts of irrigation. This can have implications outside of the HCW, by helping to reduce the over fertilization of ecosystems downstream, which can lead to eutrophication, such as with the persistent conditions seen in the Gulf of Mexico.

## Figures and Tables

**Table 1:** The linear regression analysis between significant variables and annual nitrate flux (*n* = 15). Significance levels are denoted by; *p* < 0.001 shown as *** *p* < 0.01 shown as ** *p* < 0.05 shown as * *p* < 0.1 shown as •.

Independent Variable Name	Mean	SD	Min	Max	Coefficients	*p* Value	R^2^	*n*
Precipitation Spring (mm)	304.97	94.20	131.50	448.90	2897.0	0.005 **	0.470	15
Total Irrigation from Well Water (mm)	82.35	26.90	44.48	129.21	−9108	0.015 *	0.379	15
GDD Fall/Winter Base Level 0 °C	338.54	129.14	130.15	557.75	−1696.1	0.033 *	0.303	15
Open Water (Acres)	9635	1721	6973	12,876	171.1	0.066 •	0.404	9

**Table 2:** The multiple linear regression analysis between the two significant variables of spring precipitation and annual well irrigation, and annual nitrate flux (*n* = 15). Significance levels are denoted by; *p* < 0.001 show as *** *p* < 0.01 shown as ** *p* < 0.05 shown as * *p* < 0.1 shown as •.

	Coefficients	*p* Value
Spring Precipitation (mm) (1)	7669.7	<0.001 ***
Annual Irrigation from Well Water (mm) (2)	10,769.9	0.07 •
(1) × (2)	−55.1	0.004 **
Intercept	−1,449,518	0.020 *
Model R^2^	0.858	

## Appendix A

**Table A1:** The linear regression analysis between various variables and annual nitrate flux (*n* = 15). Rows with bold text represent a variable having significant correlation with nitrate flux. Significance levels are denoted by; *p* < 0.001 shown as *** *p* < 0.01 shown as ** *p* < 0.05 shown as * *p* < 0.1 shown as •.

Independent Variable Name	Mean	SD	Min	Max	Coefficients	*p* Value	R^2^	*n*
Winter Wheat Planted (Acres)	2.36 × 10^5^	1.98 × 10^4^	1.84 × 10^5^	2.74 × 10^5^	7.356	0.179	0.134	15
Corn Planted (Acres)	1.14 × 10^5^	1.70 × 10^4^	8.47 × 10^4^	1.49 × 10^5^	−0.95	0.885	0.002	15
Soybeans Planted (Acres)	1.72 × 10^5^	3.20 × 10^4^	1.26 × 10^5^	2.26 × 10^5^	4.35	0.202	0.122	15
Alfalfa Planted (Acres)	29,992	5008.29	23,344	39,135	32.87	0.348	0.126	9
Oats Planted (Acres)	270.02	265.74	69.28	951.03	−766.4	0.236	0.193	9
Rye Planted (Acres)	71.34	82.29	1.56	217.92	2043	0.337	0.132	9
Sorghum Planted (Acres)	89,047	17,596.86	69,024	122,766	1.135	0.912	0.002	9
Sunflowers Planted (Acres)	683.40	765.25	3.78	2194.06	−168.5	0.468	0.077	9
Barley Planted (Acres)	7.74	10.10	0.67	30.98	5493	0.787	0.013	8
Fallow/Idle Cropland (Acres)	7879	10,224	2318	34,955	−15.01	0.384	0.110	9
Winter Wheat Production (BU)	2.54 × 10^7^	6.51 × 10^6^	1.84 × 10^7^	4.37 × 10^7^	−0.012	0.469	0.041	15
Corn Production (BU)	1.46 × 10^7^	7.96 × 10^6^	1.18 × 10^6^	2.53 × 10^7^	0.003	0.841	0.003	15
Soybeans Production (BU)	5.80 × 10^6^	3.65 × 106	0	1.09 × 10^7^	0.020	0.516	0.033	15
Precipitation Fall/Winter (mm)	93.61	65.35	21.90	256.20	1605.0	0.258	0.097	15
**Precipitation Spring (mm)**	**304.97**	**94.20**	**131.50**	**448.90**	**2897.0**	**0.005 ****	**0.470**	**15**
Precipitation Summer (mm)	331.68	105.52	177.80	535.10	555.1	0.601	0.022	15
Snow Fall/Winter (mm)	393.07	257.80	89.00	1012.00	251.0	0.563	0.026	15
Snow Spring (mm)	40.53	47.38	0.00	152.00	−2048.0	0.381	0.059	15
**Total Irrigation from Well Water (mm)**	**82.35**	**26.90**	**44.48**	**129.21**	**−9108**	**0.015 ***	**0.379**	**15**
**GDD Fall/Winter Base Level 0 °C**	**338.54**	**129.14**	**130.15**	**557.75**	**−1696.1**	**0.033 ***	**0.303**	**15**
GDD Spring Base Level 0 °C	1715.56	278.47	1148.15	2189.75	28.27	0.944	<0.001	15
GDD Summer Base Level 0 °C	2535.54	189.78	2128.45	2807.20	−26.24	0.965	<0.001	15
GDD Fall/Winter Base Level 10 °C	22.16	22.38	1.95	75.35	−5310.0	0.280	0.089	15
GDD Spring Base Level 10 °C	750.95	131.98	510.35	1017.55	−300.5	0.724	0.010	15
GDD Summer Base Level 10 °C	1377.58	140.51	1045.75	1612.75	−184.2	0.818	0.004	15
Grassland/Pasture (Acres)	452,543	63,233	375,554	577,197	−2.877	0.296	0.154	9
Barren Land (Acres)	38.35	18.89	10.80	67.92	5542	0.559	0.051	9
Herbaceous Wetlands (Acres)	106.88	88.75	30.01	311.34	724.6	0.722	0.019	9
Woody Wetlands (Acres)	4543	1445	2704	6493	97.91	0.424	0.093	9
**Open Water (Acres)**	**9635**	**1721**	**6973**	**12,876**	**171.1**	**0.066 •**	**0.404**	**9**
Deciduous Forest (Acres)	62,573	10,631	53,039	88,223	21.07	0.187	0.234	9
Evergreen Forest (Acres)	5.79	10.42	0.44	30.98	−13295	0.502	0.078	8
Mixed Forest (Acres)	43.26	28.05	7.98	97.60	4596	0.468	0.077	9
Developed Land—High Intensity (Acres)	303.7	30.91	250.2	335.8	5824	0.301	0.151	9
Developed Land—Medium Intensity (Acres)	1102.7	105.03	995.4	1305.9	−1548	0.354	0.123	9
Developed Land—Low Intensity (Acres)	8450	429.27	7829	8,902	−116.4	0.782	0.012	9
Developed Land—Open Space (Acres)	60,907	15,513.63	47,337	83,324	−2.325	0.842	0.006	9
